# Scientometric analysis of lipid metabolism in macrophage polarization: 2013-2023

**DOI:** 10.3389/fonc.2025.1532862

**Published:** 2025-06-19

**Authors:** Chenxiao Ye, Xiaosong Ru, Yong Guo

**Affiliations:** ^1^ The First Clinical Medical College, Zhejiang Chinese Medical University, Hangzhou, China; ^2^ Department of Oncology, The First Affiliated Hospital of Zhejiang Chinese Medical University, Hangzhou, China

**Keywords:** bibliometric analysis, macrophage polarization, lipid metabolism, tumor microenvironment, atherosclerosis

## Abstract

**Introduction:**

Macrophage polarization plays a crucial role in the development of various diseases. Simultaneously, macrophage polarization is intimately related to lipid metabolism. To assess the emerging trend of lipid metabolism during macrophage polarization, the present study was carried out.

**Methods:**

First, 472 publications that explored lipid metabolism during macrophage polarization were gathered using the Web of Science Core Collection database (WoSCC). The emerging trends were then described using CiteSpace.

**Results:**

Between 2013 and 2023, 46 countries/regions involving 268 institutions carried out studies in this field. The largest number of publications was found in China. Additionally, the most recent burst keywords included “immunometabolism”, “foam cell formation”, and “pathway”, which represented the current frontiers of research.

**Discussion:**

The detailed analysis of publications pertaining to lipid metabolism during macrophage polarization reveals the current research status and identifies its hotspots.

## Introduction

The process known as “macrophage polarization” refers to the ability of macrophages to modify their gene expression profile, metabolism, and function in response to microenvironmental stimulus (1). Numerous diseases, such as atherosclerosis (2), inflammatory bowel disease (3), and cancers (4), have been demonstrated to be significantly influenced by macrophage polarization throughout their development and progression. As known to all, macrophage lineage is characterized by heterogeneity and plasticity and undergoes phenotypic transformation under microenvironmental stimulation (5). It is well recognized that activated macrophages could be roughly categorized into two groups: M1-like macrophages, which demonstrate a pro-inflammatory phenotype, and M2-like macrophages, which demonstrate an anti-inflammatory phenotype. In detail, there are more refined subpopulation of macrophages including M1, M2a, M2b, and M2c (6, 7). Released cytokines are a major distinguishing feature between difference subpopulations of polarized macrophages. M1-like macrophages, which are classically activated, are typically interleukin (IL)-12^high^ and IL-10^low^. According to the previous research, M1-like macrophages could release pro-inflammatory factors such as IL-6, IL-23, and tumor necrosis factor−alpha (TNF-α) (1, 8). By contrast, M2-like macrophages, which are alternatively activated, are typically IL-10^high^ and IL-12^low^. The anti-inflammatory and immunoregulatory features of M2-like macrophages are well understood. In particular, M2a macrophages have been identified as wound-healing macrophages, have been reported to triggered in response to IL-4 and IL-13, and have the ability to release cytokines like transforming growth factor-beta (TGF-β) (9, 10). Furthermore, M2b macrophages are described as a “middle-of-the-road” and seem to be an exception due to their capacity to generate a number of pro-inflammatory cytokines, including IL-1β, IL-6, and IL-10 (11). Additionally, M2c and M2b macrophages are referred to as regulatory macrophages. It has been verified that M2c macrophages could produce cytokines such as IL-10 (12).

Lipids were understood to be structural elements of nuclear, organelle, and cellular membranes. To note, the importance of lipids and their metabolites in modulating macrophage polarization in multiple ways, such as regulating inflammation, phagocytosis, and response to pathogens, has come to notice in recent years. And most importantly, there is an intricate relationship between the lipid metabolism and macrophage polarization (13). Bibliometrics is one of the most effective approaches for describing the trend of a particular field of research. Additionally, it could be employed to forecast research hotspots and identify emerging research interests. Thus, we conducted a bibliometric analysis of publications that focused on the lipid metabolism during macrophage polarization issued between 2013 and 2023. This work would provide a concise summary of the research’s accomplishments.

## Methods

### Data collection

**Figure 1:**
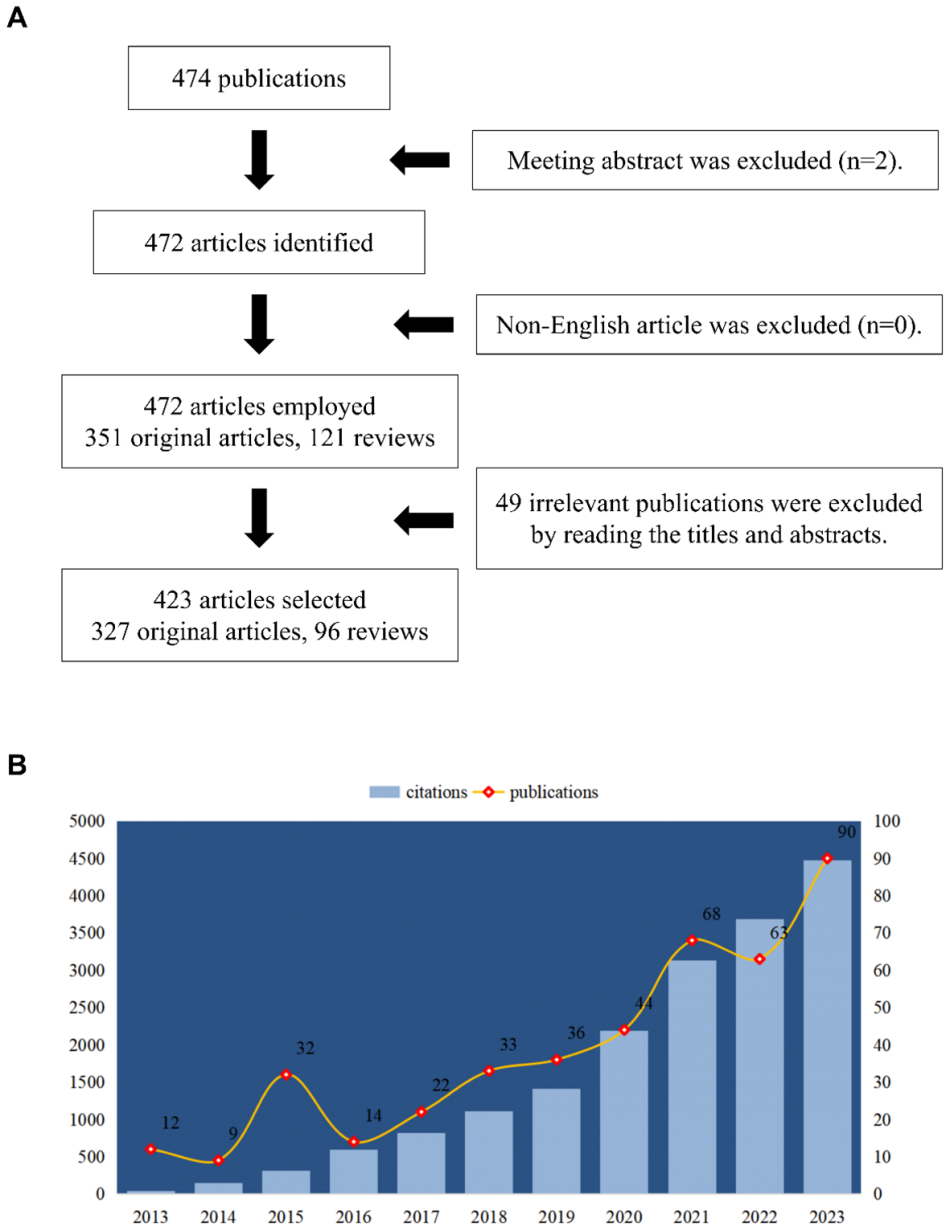
**(A)** The analytical process flowchart. **(B)** Trend of publications and citations.

The research was conducted using the Web of Science Core Collection (WoSCC). In order to prevent fluctuations in citations brought on by quick publication updates, the literature retrieval was completed in a single day (August 21, 2024). The search formula was set to TS= (macrophage polarization) and TS= (lipid metabolism). The first phase resulted in the acquisition of 474 publications (including early access) issued between 2013 and 2023. Then, 472 publications were included, comprising 351 articles and 121 review articles. Additionally, only English-language articles (n=472) were retained. In order to filter articles that were relevant to our study (lipid metabolism in macrophage polarization), we lastly reviewed the titles, abstracts, or even the whole texts of these publications. A total of 49 publications were excluded via this step. Finally, only 423 studies were enrolled for bibliometrics analysis. The detailed process was shown in **Figure 1A**. The number of publications and citations, titles, year of publication, countries/regions, affiliations, authors, journals, references, and keywords were all included.

### Statistical analysis and visualization

Before analysis, the publications are briefly described as shown in **Figure 1B**. WOSCC was employed to export all data in the model of “full record and cited references.” Chen (14) developed the bibliometric analysis tool CiteSpace. To get rid of duplicate publications, we imported the “download.txt” file into CiteSpace 6.4.R1 at first. Numerous functions, including illustrating the authors, countries/regions, institutions collaboration, co-cited references, citation burst, keywords co-occurrence, and others, can be realized by it. At the same time, we can rapidly comprehend the growth of knowledge structures, milestone articles, emerging topics, and research hotspots and frontiers thanks to its unique betweenness centrality and bursts analysis. The time slice for our analysis is set to 1 year, the g-index to *k* = 25. In addition, the parameter settings also include selecting pathfinder, pruning sliced networks, and pruning the merged network. Finally, in the country analysis, we merged the “Peoples R China” and “Taiwan.” Meanwhile, in the keyword analysis, we merged the “macrophage polarization” and “polarization.”

## Results

### The trend of publications and citations

A total of 423 articles and review articles pertaining to macrophage polarization and lipid metabolism were filtered out between 2013 and 2023. Research trends were shown in **Figure 1B**, which illustrated the yearly increase of publications and citations. In addition, as shown in **Figure 1B**, the annual publications in this area were relatively low in 2013, 2015, and 2016. And as growing interest focused on lipid metabolism and macrophage polarization, the annual outputs in this field increased steadily and sharply from 2016 to 2023. In 2023, the number of issued articles reached 90, while the citations also reached a peak of 4477. Furthermore, as revealed in **Table 1**, the highly cited article issued in 2019 entitled “*The metabolic signature of macrophage responses*.” The total citations (TC) and total citations per year (TC/Y) were 1082 and 180.33, respectively.

**Table 1:** Top 10 articles in the field.

Rank	Author	Title	Source	TC	TC/Y
1	Antonella Viola et al.	The metabolic signature of macrophage responses (2019)	Frontiers in immunology	1082	180.33
2	Stanley Ching-Cheng Huang et al.	Cell-intrinsic lysosomal lipolysis is essential for alternative activation of macrophages (2014)	Nature immunology	806	73.27
3	Thomas Weichhart et al.	Regulation of innate immune cell function by mTOR (2015)	Nature reviews. Immunology	571	57.1
4	Mario Kratz et al.	Metabolic dysfunction drives a mechanistically distinct proinflammatory phenotype in adipose tissue macrophages (2014)	Cell metabolism	566	51.45
5	Emily A Day et al.	AMPK as a therapeutic target for treating metabolic diseases (2017)	Trends in endocrinology and metabolism: TEM	441	55.13
6	Kun Liu et al.	Impaired macrophage autophagy increases the immune response in obese mice by promoting proinflammatory macrophage polarization (2015)	Autophagy	357	35.7
7	Oliver Krenkel et al.	Therapeutic inhibition of inflammatory monocyte recruitment reduces steatohepatitis and liver fibrosis (2018)	Hepatology (Baltimore, Md.)	355	50.71
8	Mireille Ouimet et al.	MicroRNA-33-dependent regulation of macrophage metabolism directs immune cell polarization in atherosclerosis (2015)	The Journal of clinical investigation	305	30.5
9	Lily Boutens et al.	Adipose tissue macrophages: going off track during obesity (2016)	Diabetologia	293	32.56
10	Alan J Mouton et al.	Obesity, hypertension, and cardiac dysfunction novel roles of immunometabolism in macrophage activation and inflammation (2020)	Circulation research	282	56.4

### The analysis of countries/regions and institutions

**Figure 2:**
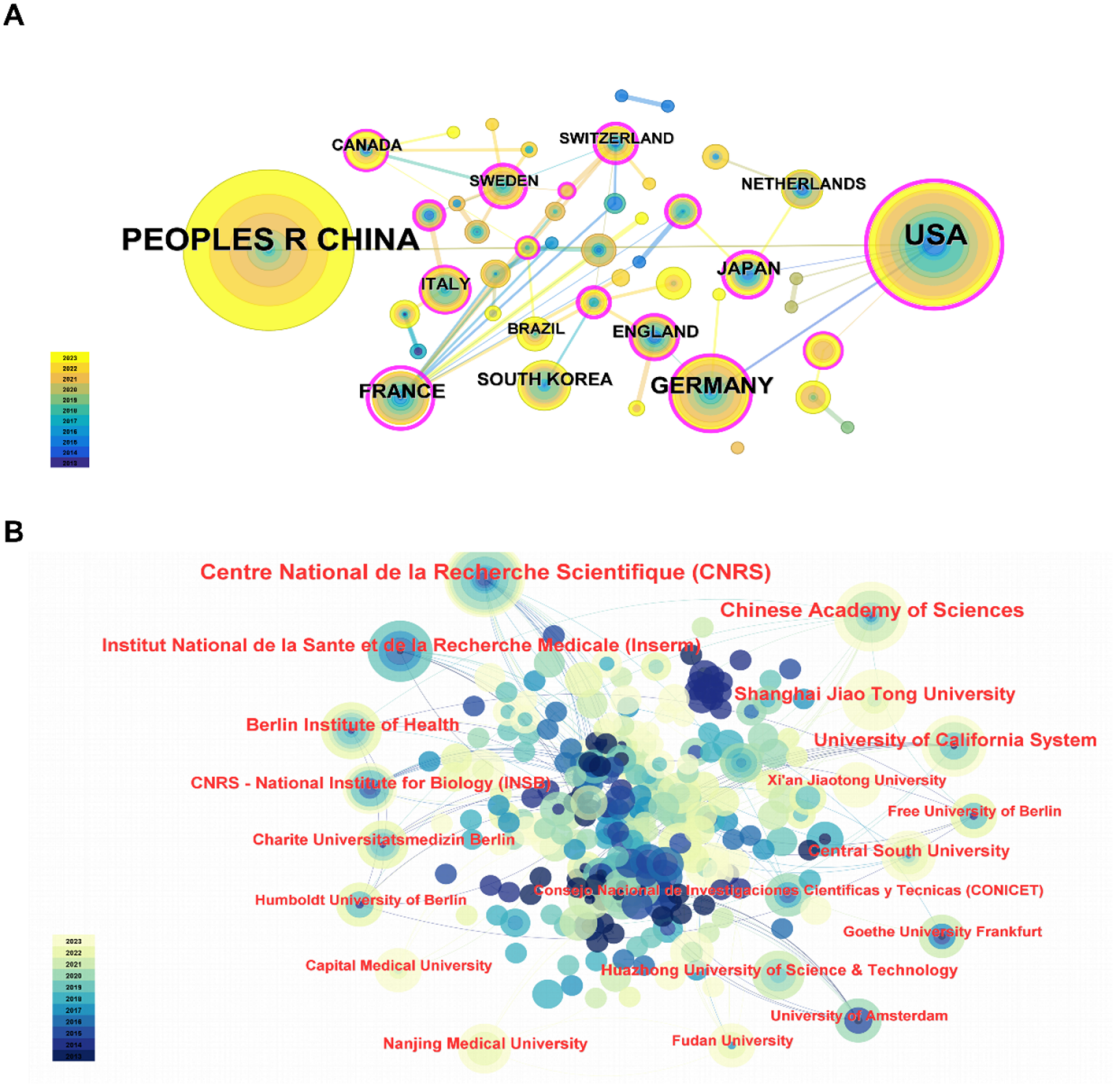
**(A)** Visual analysis of national cooperation. **(B)** Institution co-occurrence map.

Studies in this area were conducted by 46 countries/regions involving 268 institutions between 2013 and 2023. The top 10 productive countries/regions were displayed in **Table 2**. Obviously, China exhibited the most significant number of publications related to lipid metabolism and macrophage polarization (n=173), which was followed by the USA (n=114) and Germany (n=41) in second and third place, respectively. In addition, a further co-authorship network to display the cooperation among countries/regions was mapped, as shown in **Figure 2A**. Among them, China cooperates most closely with the USA, while France cooperates very frequently with other countries.

**Table 2:** Top 10 productive countries.

Rank	Country	Centrality	Year	Publications
1	PEOPLES R CHINA	0	2015	173
2	USA	0.51	2013	114
3	GERMANY	0.24	2013	41
4	FRANCE	0.87	2013	23
5	JAPAN	0.48	2013	17
6	SOUTH KOREA	0	2015	16
7	ITALY	0.16	2015	14
8	ENGLAND	0.29	2015	13
9	NETHERLANDS	0.08	2013	12
10	CANADA	0.13	2015	11

And then, **Table 3** listed the top 10 productive institutions. Among them, the Centre National de la Recherche Scientifique and the Chinese Academy of Sciences led the outputs, followed by the University of California System. In detail, the Centre National de la Recherche Scientifique has published 15 articles since 2014. And the Chinese Academy of Sciences has published 12 articles since 2015. In addition, there are 9 publications published by the University of California System from 2013. Furthermore, the co-authorship network of institutions demonstrated relatively weak collaboration among them, suggesting that cooperation among institutions needs to be further strengthened (**Figure 2B**).

**Table 3:** Top 10 productive institutions.

Rank	Institutions	Centrality	Year	Publications
1	Centre National de la Recherche Scientifique (CNRS)	0.05	2014	15
2	Chinese Academy of Sciences	0.06	2015	12
3	University of California System	0.01	2013	9
4	Shanghai Jiao Tong University	0.02	2021	9
5	Institut National de la Sante et de la Recherche Medicale (Inserm)	0.03	2013	9
6	Berlin Institute of Health	0.01	2014	8
7	Central South University	0	2017	7
8	CNRS - National Institute for Biology (INSB)	0.01	2015	7
9	Charite Universitatsmedizin Berlin	0	2014	6
10	Huazhong University of Science & Technology	0	2019	6

### Authors and co-cited authors

**Figure 3:**
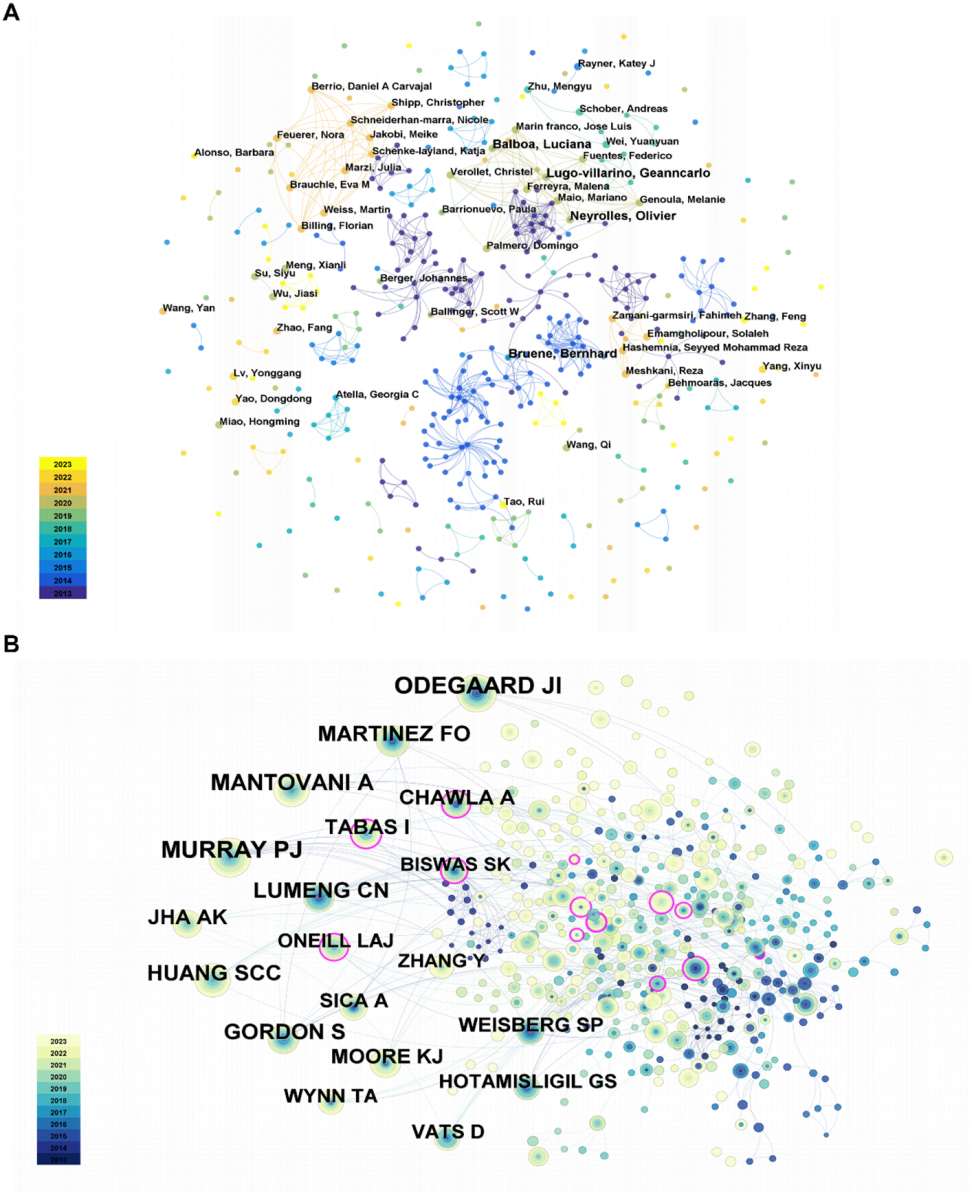
**(A)** Analysis of author’s cooperation. **(B)** Co-occurrence of cited-author.

From 2013 to 2023, a total of 425 authors carried out the studies on lipid metabolism and macrophage polarization. The authors’ cooperation network is shown in **Figure 3A**.

It is evident that individual author collaboration networks are relatively clustered into clusters, possibly due to a preference for publishing in specific research areas. At the same time, the collaborations among the author clusters also needed to be further strengthened. In addition, the top 20 highly productive authors were summarized in **Supplementary Table 1**. The writers with the most publications, as indicated in **Supplementary Table 1**, published 3 papers in related topics. These authors include Neyrolles Olivier, Lugo-villarino Geanncarlo, Balboa Luciana, and Bruene Bernhard.

Neyrolles Olivier and Lugo-villarino Geanncarlo have been producing since 2014, while Balboa Luciana and Bruene Bernhard have been publishing since 2018 and 2013, respectively.

Co-cited authors are authors who are cited in articles at the same time. To illustrate the relationships between co-cited authors, a brief network was created (**Figure 3B**). Additionally, **Table 4** included a detailed breakdown of the top 10 co-cited authors. Evidently, just 2 authors received more 80 citations, while 7 authors in total were co-cited more than 50 time. Lastly, the top three co-cited authors were MURRAY PJ, ODEGAARD JI, and MANTOVANI A. In detail, MURRAY PJ received 87 citations from 2014, ODEGAARD JI received 84 citations from 2013, and MANTOVANI A received 73 citations from 2013.

**Table 4:** Top 10 co-cited authors.

Rank	Co-cited author	Centrality	Year	Co-citation
1	MURRAY PJ	0.03	2014	87
2	ODEGAARD JI	0.09	2013	84
3	MANTOVANI A	0.07	2013	73
4	GORDON S	0.08	2013	58
5	LUMENG CN	0.06	2013	57
6	HUANG SCC	0.08	2017	56
7	MARTINEZ FO	0.08	2013	55
8	JHA AK	0.09	2017	46
9	CHAWLA A	0.17	2013	45
10	TABAS I	0.14	2015	45

### Co-citation analysis of references

**Figure 4:**
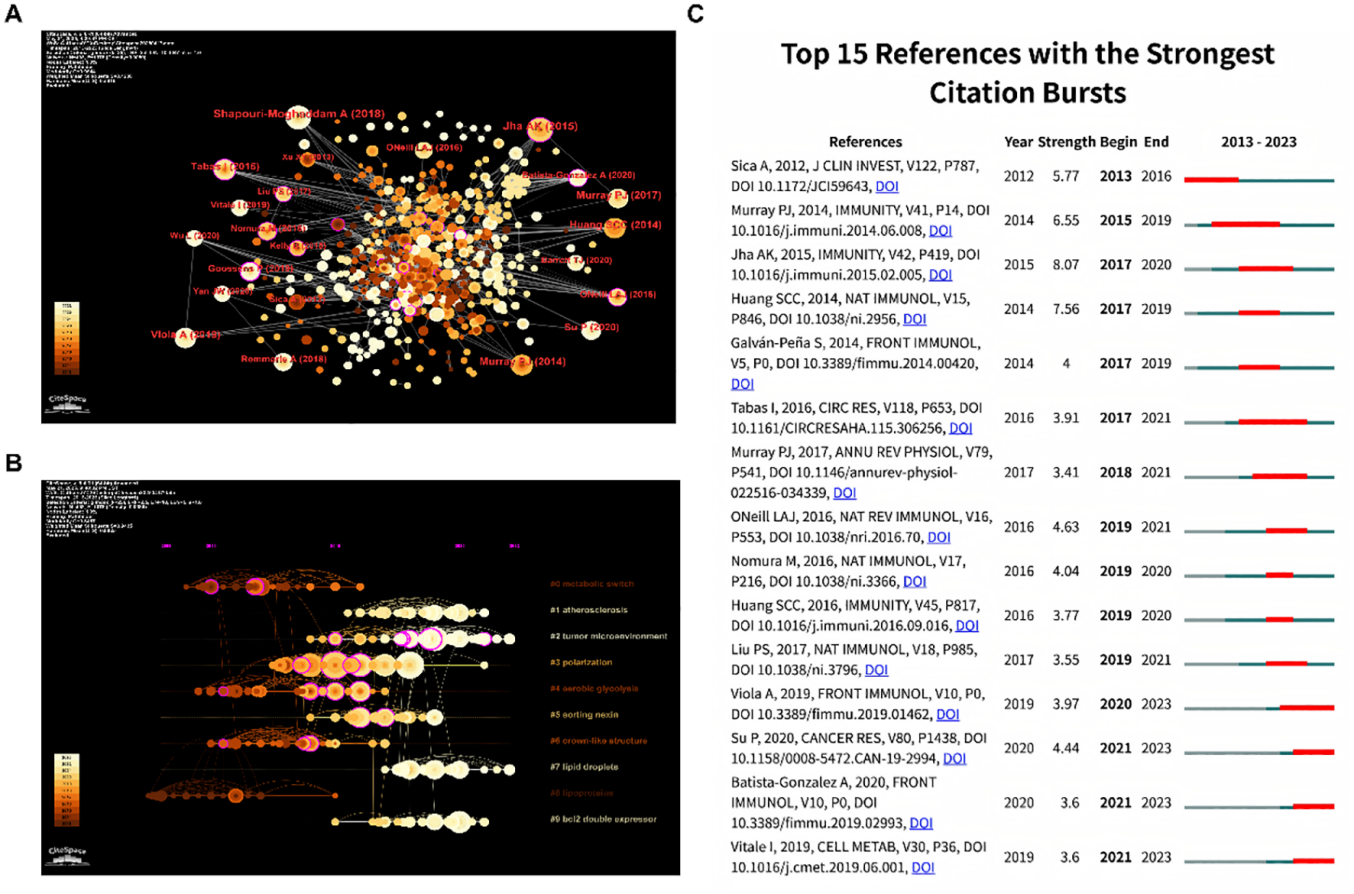
**(A)** References co-occurrence map. **(B)** References timeline view. **(C)** Burst analysis of references.

One of the CiteSpace’s most distinctive features is the co-citation network, which enables us to comprehend the patterns and development contexts in a certain subject (15). In **Figure 4A**, the colors go from darker to lighter, indicating that the cited reference is more recent. It is evident that a sizable portion of the referenced sources are recent publications, which is helpful and potent to access the cutting-edge research directions through further literature analysis. In addition, **Table 5** displayed the top 10 most cited references in detail. The most cited reference was published by Shapouri-Moghaddam A et al. (16). entitled “*Macrophage plasticity, polarization, and function in health and disease*.” In this review, the fundamental biology of macrophages, including their origin, differentiation and activation, tissue distribution, migration, and more, is covered in detail in this review. Secondly, it goes over the protective and pathogenic roles of the macrophages in both healthy and unhealthy pregnancies, atherosclerosis, fibrosis, wound healing, autoimmunity, metabolic disease and obesity, anti-microbial defense, anti-tumor immunity, and asthma and allergy. Furthermore, the reference ranked second in citations was written by Jha AK et al., which entitled “*Network integration of parallel metabolic and transcriptional data reveals metabolic modules that regulate macrophage polarization*.” The systemic alterations during murine M1-like and M2-like macrophage polarization were compiled in this article, with the former highly associated with TCA cycle fragmentation and the latter with activation of glutamine catabolism (17). In the meantime, Tabas I reviewed the latest developments on macrophage polarization and function in various atherosclerosis phases (18), showing that macrophage polarization might be crucial in every stage of this disease. Su P discovered a close connection between the fatty acid oxidation-dependent signaling pathway and human breast, colon, and prostate cancer. He suggested this pathway as a special mechanism that regulates the development and activity of protumor macrophages (19).

**Table 5:** Top 10 most cited references.

Rank	First Author	Title	Journal	No. of citations	Year	DOI
1	Shapouri-Moghaddam A	Macrophage plasticity, polarization, and function in health and disease	Nature immunology	(27)	2018	10.1002/jcp.26429
2	Jha AK	Network integration of parallel metabolic and transcriptional data reveals metabolic modules that regulate macrophage polarization	Immunity	(24)	2015	10.1016/j.immuni.2015.02.005
3	Murray PJ	Macrophage activation and polarization: nomenclature and experimental guidelines	Trends in endocrinology and metabolism: TEM	(21)	2014	10.1016/j.immuni.2014.06.008
4	Huang SCC	Cell-intrinsic lysosomal lipolysis is essential for alternative activation of macrophages	Nature immunology	(20)	2014	10.1038/ni.2956
5	Viola A	The Metabolic Signature of Macrophage Responses	Frontiers in immunology	(20)	2019	10.3389/fimmu.2019.01462
6	Murray PJ	Macrophage Polarization	Annual review of physiology	(19)	2017	10.1146/annurev-physiol-022516-034339
7	Tabas I	Macrophage Phenotype and Function in Different Stages of Atherosclerosis	Circulation research	(18)	2016	10.1161/CIRCRESAHA.115.306256
8	Su P	Enhanced Lipid Accumulation and Metabolism Are Required for the Differentiation and Activation of Tumor-Associated Macrophages	Cancer research	(16)	2020	10.1158/0008-5472.CAN-19-2994
9	Remmerie A	Macrophages and lipid metabolism	Cellular immunology	(14)	2018	10.1016/j.cellimm.2018.01.020
10	Goossens P	Membrane Cholesterol Efflux Drives Tumor-Associated Macrophage Reprogramming and Tumor Progression	Cell metabolism	(14)	2019	10.1016/j.cmet.2019.02.016

Furthermore, the timeline map (**Figure 4B**) was drawn after clustering and named with keywords. Additionally, each cluster’s progress might be more clearly presented. The upcoming trends were shown by clusters 1, 2, 7, and 9. And to note, investigations on the tumor microenvironment in particular are probably going to be an increasingly prevalent topic. Besides, the dense lines between Cluster 1 and Cluster 4 highlight the potential role of aerobic glycolysis in the development of atherosclerosis. Meanwhile, the lines between Cluster 6 and Cluster 8 were also relatively dense, which suggests a close relationship between crown-like structure and lipoprotein. To note, researchers focusing on tumor microenvironment and atherosclerosis are encouraged to enhance interdisciplinary collaboration.

Moreover, a visual map was further constructed to better illustrate the reference burst trend (**Figure 4C**). According to burst analysis, some authors have had a comparatively high number of citations in recent years. For example, the Metabolic Signature of Macrophage Responses was detailed in Viola A’s article, “The Metabolic Signature of Macrophage Responses,” which summarized that M1-like and M2-like macrophages are distinguished by particular pathways that control the metabolism of lipids and amino acids and influence their responses. All of these metabolic adaptations are useful to maintain their polarization in particular situations and to support macrophage activities (20). Besides, the study by Jha AK et al. (2015) got the greatest burst strength (8.07).

### Analysis of keywords

**Figure 5:**
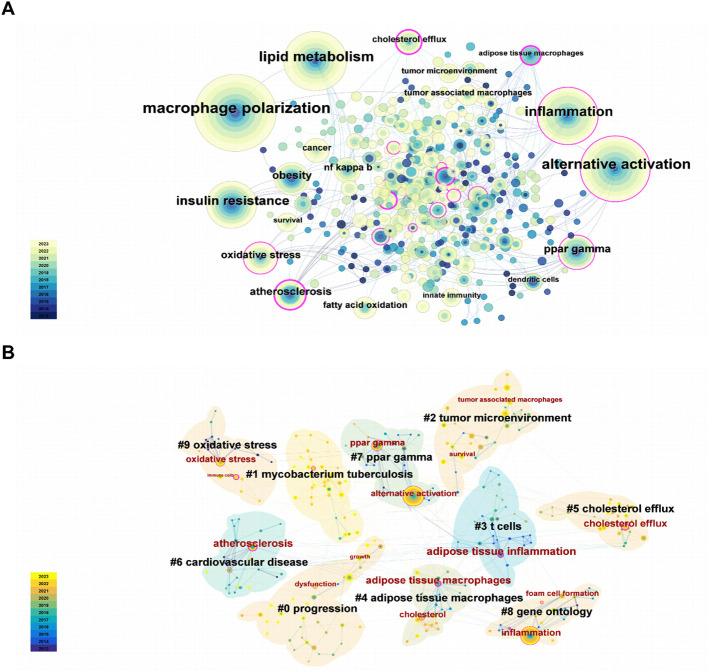
**(A)** Keyword co-occurrence map. **(B)** Cluster analysis of keyword.

First of all, a total of 19 keywords were identified as occurring more than 10 times, which have been illustrated in **Figure 5A**. As illustrated in **Table 6**, “alternative activation,” “inflammation,” “insulin resistance,” and “ppar gamma” were the keywords that appeared most frequently for pathological processes in this field, except for the topic terms of “macrophage polarization” and “lipid metabolism.” And interestingly, we discovered the presence of some keywords (innate immunity and dendritic cells), which may indicate that lipid metabolism mediates close communication between immune cells.

**Table 6:** Top 20 keywords.

Rank	Frequency	Centrality	Keywords	Rank	Frequency	Centrality	Keywords
1	179	0.05	macrophage polarization	11	17	0.05	fatty acid oxidation
2	129	0.13	alternative activation	12	16	0	cancer
3	106	0.07	lipid metabolism	13	15	0.23	cholesterol efflux
4	99	0.16	inflammation	14	13	0.05	tumor associated macrophages
5	73	0.08	insulin resistance	15	12	0.02	tumor microenvironment
6	38	0.11	ppar gamma	16	10	0.06	innate immunity
7	34	0.07	obesity	17	10	0.06	dendritic cells
8	30	0.35	atherosclerosis	18	10	0.21	adipose tissue macrophages
9	28	0.13	oxidative stress	19	10	0.06	survival
10	20	0.08	nf kappa b	20	9	0.08	hepatic steatosis

Moreover, keywords after clustering help to characterize the distribution of research content on a specific topic (**Figure 5B**). This led to the creation of a visual representation of keyword co-occurrence clusters. First of all, the cluster’s mean silhouette value was 0.9062 and its modularity Q was 0.7696, respectively, ensuring the high standards of this analysis. Unquestionably, the largest cluster was undoubtedly cluster #0, which was named “progression,” followed by “mycobacterium tuberculosis,” “tumor microenvironment,” “t cell,” “adipose tissue macrophage,” “cholesterol efflux,” and so on. Furthermore, specific representative nodes of each cluster were labeled. Interestingly, tumor microenvironment was the designation given to cluster #2, which included nodes recognized as survival and tumor-associated macrophages. In addition, t cells was the label given to cluster #3, which included nodes recognized as adipose tissue inflammation.

**Figure 6:**
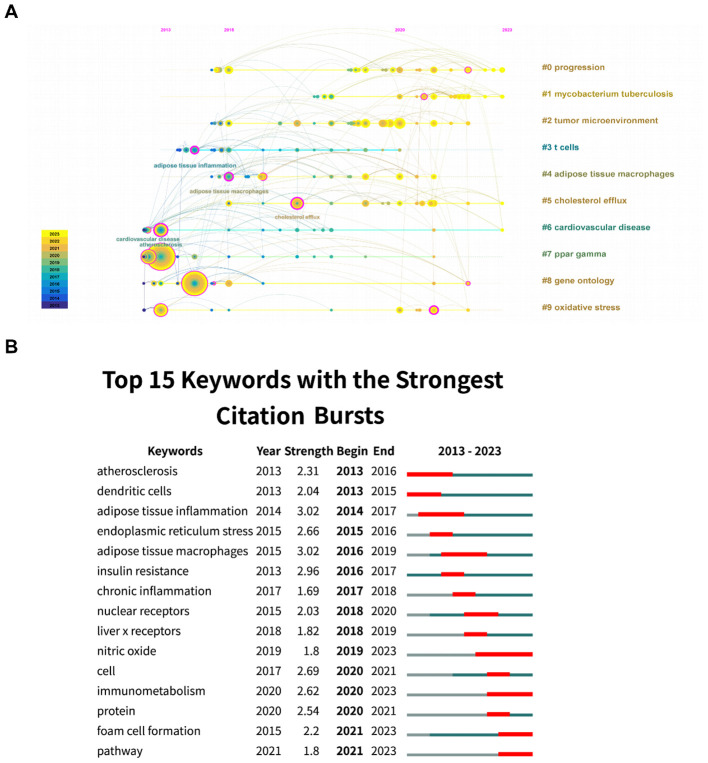
**(A)** Keyword timeline map. **(B)** Burst analysis of keywords.

From a timeline perspective, keywords were shown in **Figure 6A** based on their year of appearance. We performed further analysis of the burst of keyword citations. As illustrated in **Figure 6B**, “atherosclerosis” got the highest strength. Besides, “nitric oxide” had experienced the most prolonged bursts lasting over four years (2019-2023). Additionally, the most recent burst keywords included “immunometabolism,” “foam cell formation,” and “pathway,” which might represent the current frontiers of research.

### The effect of certain metabolites on macrophage polarization

Based on the selected literature, we further analyzed the different effects of several metabolites on macrophage polarization and the important enzymes involved in this process (**Table 7**), in order to thoroughly elucidate the role of lipid metabolism in macrophage polarization. As illustrated in **Table 7**, some lipid metabolites, such as palmitic acid, docosahexaenoic acid, prostaglandin E2, arachidonic acid, maresin 1, lysophosphatidylcholine, prostaglandin E3, and 5-aminolevulinate, have a significant impact on macrophage polarization and alter the expression levels of certain proteins. Additionally, it is suggested that PPAR-γ, OXPHOS, kinase A, and CX3CR1 are important enzymes in this process. To note, PPAR-γ seems to be a key enzyme of particular interest.

**Table 7:** The effect of part metabolites generated in lipid metabolism on macrophage polarization.

Metabolites	Generation Pathway	Key proteins	Effect on Macrophages	
Palmitic acid	Saturated fatty acids	PPAR-γ	M1-polarized macrophages↑	iNOS, TNF-α, and IL-6↑
Docosahexaenoic acid	Polyunsaturated fatty acids	PPAR-γ	M2-polarized macrophages↑	MRC2, IL-10↑
Prostaglandin E2	Derivative of fatty acids	PPAR-γ, OXPHOS	M2-polarized macrophages↑	IL-1RA, CD209, CD206↑CCR7, and CD69↓
Arachidonic acid	Arachidonic acid metabolism	PPAR-γ, OXPHOS	M2-polarized macrophages↓	IL-4, TARC, CD209, CD206, and CCR7↓
Maresin 1	Fatty acid	PPAR-γ	M2-polarized macrophages↑	CD206, Arg-1, IL-4, and IL-10↑
			M1-polarized macrophages↓	iNOS, TNF-α, and IL-6↓
Lysophosphatidylcholine	lysophospholipid	PPAR-γ	M2-polarized macrophages↑	Arg-1, MR1, CHI3l3, IL-10, and TGF-β↑
Prostaglandin E3	Derivative of fatty acids	kinase A	M2-polarized macrophages↑	CD204, CD163, TGF-β, IL-10, CCL17, CD206↑
			M1-polarized macrophages↓	iNOS, TNF-α, CXCL3, IL6, CXCL9, and CCR7↓
5-aminolevulinate	Derivative of glycine	CX3CR1	M2-polarized macrophages↑	CD206, IL-10, and Arg1↑
			M1-polarized macrophages↓	iNOS and Il-1β↓

## Discussion

By analyzing the main areas of knowledge and emerging trends of lipid metabolism during macrophage polarization, we have gained some meaningful information. First of all, this study covered 423 publications in all. What’ more, annually increasing publications and citations of them were observed with time. In addition, not only is China the most productive country, one of the most productive organizations (Chinese Academy of Sciences) is also located in China. To note, according to the timelines of references, macrophage polarization in tumor microenvironment and atherosclerosis has lately attracted researchers’ close attention. Meanwhile, according to the timelines of keywords, macrophage polarization in mycobacterium tuberculosis and tumor microenvironment has lately attracted researchers’ close attention. Taken together, tumor microenvironment is an attractive and potential study subject in related academic fields.

However, according to the timelines of references and keywords, the lines between tumor microenvironment and other clusters are relatively lacking. Therefore, researchers focusing on tumor microenvironment are encouraged to enhance interdisciplinary collaboration. Citation burst analysis is a tool visualizing the emerging and potential research trends in a field. To investigate possible research directions for research in areas pertaining to the tumor microenvironment, citation burst analysis were employed. Citation burst analysis of keywords revealed that keywords of nuclear receptors, liver x receptors, nitric oxide, cell, immunometabolism, protein, foam cell formation, and pathway burst from 2018 to 2023. Taken together, the emerging and potential research trends are focused on study of mechanisms at the cellular level. Furthermore, in reference citation burst analysis, the most substantial citation burst publication was issued by Antonio Sica et al. in 2012, which reviewed the molecules and mechanisms underlying macrophage polarization and plasticity (21). The second citation burst review article was published by Peter J Murray et al. in *Immunity* in 2014 (22). Their team clarified the origin of macrophages and proposed a common definition of macrophage activation in that review. And the third citation burst publication is produced by Abhishek K Jha et al. in 2015. In this study, an integrated high-throughput transcriptional-metabolic profiling and analysis workflow was used to describe systemic alterations that occurred throughout the polarization of M1-like and M2-like murine macrophages. More detailed, glutamine catabolism and UDP-GlcNAc-associated modules are closely related with the occurrence of M2-like macrophages. Accordingly, M2-like macrophages and the chemokine CCL22 production were reduced by glutamine deprivation or N-glycosylation inhibition. Remarkably, metabolites could play a significant role in macrophage polarization. Therefore, metabolite-based unfolding of macrophage polarization at the cellular level is a potentially valuable research direction. Therefore, we summarized the metabolites generated in lipid metabolism, and illustrated its effect on macrophage polarization as below.

According to the issued publications, metabolites generated in lipid metabolism could impact on the fate of macrophage polarization (**Table 7**). According to investigation by Hui-Min Wu et al., certain lipids may cause macrophage polarization, which then affects hepatocyte lipid metabolism directly. In detail, their team confirmed that palmitic acid (PA), as one of the saturated fatty acids, induced M1-like macrophage polarization with upregulation of iNOS, TNF-α, and IL-6. While docosahexaenoic acid (DHA), as one of the polyunsaturated acids, induced M2-like macrophage polarization with upregulation of MRC2 and IL-10 (23). Miao Xu et al. have reported that arachidonic acid could prevent M2-like macrophage polarization. But interestingly, by elevating the relative expression of IL-1RA, CD209, and CD206 and decreasing the relative expression of CCR7, and CD69, prostaglandin E2 (PGE2), a metabolite of arachidonic acid, inversely promotes M2-like macrophage polarization (24). To note, PPARγ links these two processes by regulating oxidative phosphorylation (OXPHOS). In 2022, Dongdong Yao et al. published an article in *Biomaterials advances* (25). They summarized that MaR1 is strongly capable of triggering peroxisome proliferator-activated receptor-γ (PPAR-γ) activation and M2-like macrophage polarization with upregulation of CD206, Arg-1, IL-4, and IL-10. Besides, MaR1 had a strong capability and performance in depressing M1-like macrophage polarization with downregulation of iNOS, IL-6, and TNF-α. Leonardo Santos Assunção et al. have demonstrated the immunomodulatory property of lysophosphatidylcholine (LPC) (26). In detail, their team summarized that lysophosphatidylcholine generated from schistosomal is strongly linked to M2-like macrophage polarization accompanied by increases in Arg-1, MR1, CHI3l3, IL-10, and TGF-β. Furthermore, the effects of the above metabolites on macrophage polarization are largely dependent on PPARγ activity.

In addition, Jing Cui et al. (27) summarized that Prostaglandin E_3_ (PGE_3_), a fatty acid derivative, may suppress M1-like macrophage polarization while promoting M2a macrophage polarization. More detailed, PGE_3_ could potently increase the relative expression of CD204, CD163, TGF-β, IL-10, CCL17, and CD206, while decrease the relative levels of iNOS, TNF-α, CXCL3, IL6, CXCL9, and CCR7. Guanghui Jin et al. reported that 5-aminolevulinate (5-ALA) triggered M2-like macrophage polarization and upregulated CD206, IL-10, and Arg1. Simultaneously, it suppressed M1-like macrophage polarization and downregulated iNOS and Il-1β (28). Notably, CX3CR1 is the key enzyme in this process.

Studies mentioned above suggested the potential value of lipid metabolism and macrophage polarization in cancer research, especially tumor microenvironment. To note, PPAR-γ seemed to be a potential target. Accordingly, a recent publication by Vikash Kansal et al. (29) focused on the effect of statin drugs on immune checkpoint blockade in head and neck squamous cell carcinoma (HNSCC). In this study, stains were experimentally shown to enhance responses to immunotherapies for HNSCC, which encourages further investigation into the application of medications that target lipid metabolism in combination with chemotherapy, radiotherapy, and other clinical treatment in modern cancer management.

What’ more, specific representative nodes of each cluster were labeled in the analysis of keywords. Surprisingly, tumor microenvironment was the label given to cluster #2, which included nodes recognized as survival and tumor-associated macrophages. To note, survival data is essential for studying tumors. Therefore, it is worthwhile to investigate not just lipid metabolism and macrophage polarization in the tumor microenvironment, but also survival in combination with these factors since it may lead to new discoveries. Additionally, there is a dearth of research in this area. According to Jake et al., the plasma metabolome was considerably changed by acute-on-chronic liver failure, specifically in the areas of energy metabolism, oxidative stress pathways, steroid hormones, and membrane lipid metabolism. Several metabolites were substantially linked to pre-ACLF in the non-ACLF group and/or 90-day mortality in the ACLF group. Furthermore, they developed assays for HBV-related ACLF with greater accuracy than current techniques, based on novel metabolite biomarkers (30). The esteemed journal Journal of Hepatology published this finding. Taken together, combining lipid and macrophage metabolism might be useful for tumor clinical research.

Based on bibliometrics analysis, we have provided a relative comprehensive summary of lipid metabolism in macrophage polarization, and we look forward to further trends in this field. However, there are still several limitations in our study due to the nature of bibliometrics. To begin, despite our effects to included publications from WoSCC, we have missed some articles that are not included in WoSCC. Second, bibliometrics analysis was carried out using natural language processing and machine learning, which may cause some degree of bias. Lastly, we focused on only one specific area in this study, which may have led to a lack of a macroscopic perspective on the overall trend of research on macrophage polarization.

## Data Availability

The original contributions presented in the study are included in the article/**Supplementary Material**. Further inquiries can be directed to the corresponding author.

## Supplementary material

Supplementary Table 1 Top 20 productive authors.
