# Supplementary material for: Scientometric analysis of lipid metabolism in macrophage polarization: 2013-2023
Source: Front Oncol. 2025 Jun 19;15:1532862. doi: 10.3389/fonc.2025.1532862 (PMC12222308; doi:10.3389/fonc.2025.1532862)
Supplement: Supplementary Table 1 — Top 20 productive authors. [file Table1.docx]

**Supplementary Table 1.** Top 20 productive authors.

| Rank | Count | Year | Authors | Rank | Count | Year | Authors |
| --- | --- | --- | --- | --- | --- | --- | --- |
| 1  2  3  4  5  6  7  8  9  10 | 3  3  3  3  2  2  2  2  2  2 | 2014  2014  2018  2013  2020  2021  2021  2020  2021  2021 | Neyrolles Olivier  Lugo-villarino Geanncarlo  Balboa Luciana  Bruene Bernhard  Su Siyu  Hashemnia Seyyed Mohammad Reza  Schenke-layland Katja  Fuentes Federico  Meshkani Reza  Emamgholipour Solaleh | 11  12  13  14  15  16  17  18  19  20 | 2  2  2  2  2  2  2  2  2  2 | 2021  2021  2015  2020  2020  2021  2019  2016  2015  2021 | Wang Yan  Weiss Martin  Rayner Katey J  Maio Mariano  Wu Jiasi  Billing Florian  Behmoaras Jacques  Atella Georgia C  Ballinger Scott W  Shipp Christopher |
